# Non-HDL-C and LDL-C/HDL-C are associated with self-reported cardiovascular disease in a rural West African population: Analysis of an array of lipid metrics in an AWI-Gen sub-study

**DOI:** 10.1371/journal.pone.0278375

**Published:** 2022-12-07

**Authors:** Godfred Agongo, Frederick Raal, Engelbert A. Nonterah, Cornelius Debpuur, Abraham R. Oduro, Michèle Ramsay, Nigel J. Crowther

**Affiliations:** 1 Department of Biochemistry and Forensic Sciences, School of Chemical and Biochemical Sciences, C.K Tedam University of Technology and Applied Sciences, Navrongo, Ghana; 2 Navrongo Health Research Centre, Ghana Health Service, Navrongo, Ghana; 3 Division of Endocrinology and Metabolism, Faculty of Health Sciences, University of the Witwatersrand, Johannesburg, South Africa; 4 Julius Global Health, Julius Center for Health Sciences and Primary Care, University Medical Centre Utrecht, Utrecht University, Utrecht, The Netherlands; 5 Research and Development Division, Ghana Health Service, Accra, Ghana; 6 Sydney Brenner Institute for Molecular Bioscience, Faculty of Health Sciences, University of the Witwatersrand, Johannesburg, South Africa; 7 Division of Human Genetics, National Health Laboratory Service and School of Pathology, Faculty of Health Sciences, University of the Witwatersrand, Johannesburg, South Africa; 8 Department of Chemical Pathology, National Health Laboratory Service and School of Pathology, Faculty of Health Sciences, University of the Witwatersrand, Johannesburg, South Africa; University of Toronto, CANADA

## Abstract

Few studies have compared the utility of serum levels of lipid fractions in cardiovascular disease (CVD) risk assessment in sub-Saharan Africa (SSA). The current study interrogated this question among men and women aged 40–60 years in rural northern Ghana. This was a cross-sectional study in which data was collected on socio-demography, behaviour, health history, anthropometry and lipid levels. Adjusted multivariable logistic regression models were used to assess the association of various lipid metrics with CVD. All tests were considered statistically significant at P<0.05. Data were available for 1839 participants. The prevalence of self-reported CVD was 1.6% (n = 29). Non-HDL-C (median (interquartile range): 2.4 (1.9–3.0) vs 2.0 (1.6–2.5) mmol/L; P = 0.009), LDL-C/HDL-C (1.8 (1.4–2.4) vs 1.5 (1.1–2.6); P = 0.019) and TC/HDL-C (3.3 (2.9–3.9) vs 2.9 (2.4–3.5); P = 0.003) were all significantly higher in participants with self-reported CVD compared to those without. However, after adjusting for socioeconomic status (SES) and meals from vendors in a logistic regression model, only non-HDL-C (odds ratio [95% CIs]): (1.58 [1.05, 2.39]), P = 0.029 and LDL-C/HDL-C levels (odds ratio [95% CIs]): (1.26 [1.00, 1.59]), P = 0.045 remained significantly associated with self-reported CVD. While our findings suggest non-HDL-C and LDL-C/HDL-C measures may be appropriate biomarkers for assessing CVD risk in this population, further studies using established clinical endpoints are required to validate these findings in sub-Saharan Africans.

## Background

The burden of cardiovascular disease (CVD) is increasing globally and in sub-Saharan Africa (SSA). Thus, CVD almost doubled from 271 million in 1990 to 523 million in 2019. Within the same period the number of deaths increased from 12 million to 18.6 million [1]. It is estimated that CVD accounts for about 30% of global deaths with 80% of CVD deaths occurring in low- and middle-income countries including SSA [2]. It is therefore important to identify reliable indicators that can be used to assess CVD risk in SSA. Unfavorable lipid markers including total cholesterol (TC), low density lipoprotein cholesterol (LDL-C), high density lipoprotein cholesterol (HDL-C) and triglycerides (TG), are used as indicators in the assessment of CVD risk with recent cardiovascular-related guidelines also including non-high density lipoprotein cholesterol (non-HDL-C) as a therapeutic target [3, 4]. Although all these markers have been considered as predictors of CVD risk [4], studies in non-African populations have shown that LDL-C [5] and non-HDL-C [6] are more reliable markers of CVD risk. Thus, genetic, clinical and epidemiologic studies have shown that LDL-C is a principal cause of atherosclerotic cardiovascular disease [7] and remains the primary target of therapy [8]. Non-HDL-C, which is the difference between TC and HDL-C [9], is a proxy measure of the total atherogenic apolipoprotein B (apo B) containing lipoproteins, including very low density lipoprotein cholesterol (VLDL-C), intermediate lipoprotein cholesterol (IDL-C), chylomicrons and VLDL-C remnants, and this may explain its strong association with CVD risk [10].

Other lipid metrics that have been reported as CVD risk markers include LDL-C/HDL-C [11], TC/HDL-C [12] and TG/HDL-C ratios [13]. Studies have suggested that these lipid ratios are more accurate markers of CVD risk than LDL-C or HDL-C in some populations [14]. Similarly, it has been suggested that remnant cholesterol, which is the cholesterol content of intermediate-density lipoproteins (IDL), very-low-density lipoproteins (VLDL) and chylomicron remnants, is associated with an increased risk for ischaemic heart disease [15] and has been linked to CVD risk even when LDL-C is low [16].

However, there is still controversy as to whether LDL-C, non-HDL-C, TG, TC, the proatherogenic lipid ratios (TG/HDL-C, TC/HDL-C, and LDL-C/HDL-C) or remnant cholesterol is the most accurate marker of CVD. While reports of studies in non-African populations have suggested that the predictive value of non-HDL-C is similar to LDL-C [17, 18] others have argued that non-HDL-C is a better marker of CVD risk [19, 20]. Similarly, studies in non-African populations have also suggested that the proatherogenic lipid ratios are superior to conventional lipid parameters (LDL-C, HDL-C, TG and TC) for predicting arterial stiffness [21], which is associated with progression to atherosclerosis [22]. The paucity of data on the comparative CVD risk predictive values of LDL-C, non-HDL-C, TG, TC, proatherogenic lipid ratios and remnant cholesterol, in SSA populations, calls for the need to evaluate the predictive ability of these metrics. Furthermore, the prevalence of CVDs is increasing in SSA [23] and therefore there is an urgent need to identify the most suitable disease markers in these under-studied populations. It has been suggested that low serum HDL-C level is not a reliable predictor of CVD risk [24, 25]; however, data on the predictive value of low HDL-C in black Africans is lacking. It is interesting to note that a recent study performed in the Kassena-Nankana districts (KNDs) of northern Ghana demonstrated a high (60.3%) prevalence of low HDL-C [26]. This raises the need to evaluate its predictive value in CVD risk assessment in this population since HDL-C levels are confounded by several factors including its positive correlation with saturated fats [27]. Further to this, resource limitations particularly in SSA call for the need to identify the lipid components that best predict CVD risk so as to minimize population screening costs.

The current study therefore investigated the association of various lipid metrics with self-reported CVD. The novelty of this study is in the use of a wide array of lipid metrics in the investigation of CVD in a large SSA population in which data is available on CVD endpoints. Particularly, this is the first study to carry out such analyses in a rural, adult population resident in Ghana which is known to have a high prevalence of low HDL-C [26]. In addition, a recent meta-analysis has demonstrated a high prevalence of dyslipidaemia across the African continent [28] and this highlights the importance of determining the association of lipid levels with CVD in African populations.

## Methods

### Study design

This was a population based cross-sectional study that was conducted as part of the Africa Wits-INDEPTH Partnership for Genomic studies (AWI-Gen) project from 2013 to 2017 under the broader Human Heredity and Health in Africa (H3Africa) initiative [29]. The study was conducted in accordance with the STROBE (STrengthening the Reporting of OBservational studies in Epidemiology) guidelines for the presentation of reports of cross sectional studies [30].

### Study setting

The study was conducted in the two Kassena-Nankana districts (KNDs) (Kassena-Nankana east and west) of northern Ghana that share a border with southern Burkina Faso. The two districts are covered by the Navrongo Health and Demographic Surveillance System (NHDSS) which has categorized the area into five zones according to the geographical cardinal points (east, west, north, south and central zone). Each zone is further divided into clusters. The study setting is mainly rural with mostly agricultural activities and covers a total land area of approximately 1675 km^2^ and with an estimated population size of 167500 people [31].

### Study population

The study population consisted of men and women aged 40–60 years who were resident within the community for at least ten years and agreed by written informed consent to participate in the study. Pregnant women and individuals who could not stand upright were excluded because their weight and standing height could not be accurately measured. Participants were selected using stratified random sampling from the two KNDs of northern Ghana. Four zones (east, west, north and south) of the KNDs were first selected and from each of these zones twenty five clusters were randomly sampled using the Navrongo Health and Demographic Surveillance System (NHDSS) [31]. A list of 2200 men and women including 10% for non-response or refusal was generated from the sampled clusters. The sample size in each cluster was proportional to its population. Individuals who agreed to participate in the study provided informed consent and were assigned unique identification numbers to ensure anonymity [26, 29]. A sample size of 2019 was recruited into the study but a complete case analysis was conducted using an analytical dataset of 1839.

### Sample size determination

This study was 99.99% powered to detect dyslipidaemia with a minimum sample size of 1441 at an acceptable margin of 5% assuming a population size of about 165000 in the KNDs [31] and considering the prevalence of dyslipidaemia, as defined by low HDL-C, as 60.30% [26]. The sample size was determined using epi info TM software version 7.2.2.16 [32].

### Data collection

Potential participants in the selected sample were invited to a common venue for recruitment. Following community engagement and informed consent, data including age, sex, diet (vegetable and fruit intake, and vendor meals consumed), medication use, physical activity, smoking, socio-economic status (SES), previous congestive heart failure, myocardial infarction and stroke were collected using an interview-administered structured questionnaire. Standing height of participants was measured using a Harpenden stadiometer (Holtain, Crymych Wales) fixed to a wall while weight was measured using a weighing scale (Kendon Medical, South Africa). Waist and hip circumference of participants in light clothes were measured using a stretch-resistant tape measure (SECA, Hamburg, Germany). Blood pressure was measured using a digital sphygmomanometer (Omron M6, Omron, Kyoto, Japan) with the measurement taken thrice at two-minute intervals between each measure. The systolic blood pressure (SBP) and the diastolic blood pressure (DBP) were calculated using the means of the last two measurements [33]. Visceral adipose tissue thickness (VAT) and subcutaneous adipose tissue thickness (SAT) were measured twice using a LOGIQ e ultrasound system (GE, Healthcare, CT, USA) and their mean values calculated. All data was entered into a paper response form and captured into the REDCap platform [34]. As part of the quality control process 10% of all data entries were checked for data entry consistency and all missing variables were noted [26]. Details of data collection for age, sex, physical activity, diet, cigarette smoking and anthropometric measures (weight, height, waist and hip circumference) were previously described [26].

### Biomarker analysis

Fasting blood glucose, LDL-C, HDL-C, TC and TG were all measured directly using an automated chemistry analyzer (Randox RX Daytona+, Crumlin, Northern Ireland) as described elsewhere [26, 29]. Non-HDL-C was calculated by subtracting HDL-C from TC [9]. The fasting proatherogenic lipid ratios, i.e. TG/LDL-C, TC/HDL-C and LDL-C/HDL-C, were calculated. Remnant cholesterol was calculated by subtracting the sum of LDL-C and HDL-C from TC (TC-[LDL-C + HDL-C]) [15, 33]. To ensure quality control a random selection of 150 samples were analyzed in duplicate for glucose and the lipid fractions to ascertain the coefficient of variation (CV) of the assay.

### Ethics approval and consent to participate

This was a sub-study of the AWI-Gen (Africa Wits-INDEPTH Partnership for Genomic Research) project that was approved by the Human Research Ethics Committee (HREC) of the University of the Witwatersrand (ID No: M12109), the Ghana Health Service Ethics Review Committee (ID No: GHS-ERC:05/05/2015) and the Navrongo Institutional Review Board (ID No: NHRCIRB178). The study was conducted in accordance with United States federal code of ethics. Community engagement was carried out in the communities where participants were sampled. Individual broad informed consent, evidenced by a thumb-printed or signed informed written consent form witnessed by a researcher, was sought from participants before being recruited into the study.

### Definitions

Congestive heart failure, myocardial infarction (MI) and stroke were self-reported incidents prior to the time of recruitment and CVD was then defined as a self-reported incident of congestive heart failure, stroke or MI. Diet was determined by the average number of self-reported servings of food prepared by street vendors (the main commercial source of prepared food in this geographical area) per month, average number of self-reported consumption of fruits or vegetables per week. Smoking was defined as being a current smoker or non-smoker. High SES was defined as the fourth and fifth quintiles that were derived from the scores developed from principal components computed using household assets (http://indepth-network.org/resources/indepth-health-equity-tool-measuringsocio-economic-status). Physical activity was determined using the Global Physical Activity Questionnaire (GPAQ) [35]. Moderate to vigorous-intensity physical activity (MVPA) was defined as minutes of physical activity per week (min/week). Low physical activity was defined as MVPA<150min/week while normal/high physical activity defined as MVPA≥150min/week. Body mass index (BMI) was computed as weight (kg)/height^2^ (m^2^) with obesity as BMI≥30kg/m^2^ [36]. Hypertension was defined as SBP>140mmHg and/or DBP>90mmHg or self-reported treatment using anti-hypertensive medication [37]. High non-HDL-C was defined as non-HDL-C>3.4mmol/l, low HDL-C level as <1.0mmol/l for men and <1.2mmol/l for women, high LDL-C level as >3.0mmol/l and high TG was defined as >1.7mmol/l [38]. High TC was defined as TC>5.0mol/l [39]. High proatherogenic lipid ratios associated with CVD risk were defined as follows: LDL-C/HDL-C> 4:1 [16], TG/HDL-C> 3:1 and TC/HDL-C> 5:1 in men and 4:1 in women [40]. High remnant cholesterol was defined as remnant cholesterol> 0.80mmol/l [41].

### Statistical analyses

Data analyses were performed using STATA version 14.2 (StataCorp, College Station, Texas, US). Continuous variables were skewed and were presented as medians with interquartile ranges (IQR) and compared between individuals with and without self-reported CVD using the Mann Whitney *U* test while categorical variables were compared between those with and without self-reported CVD using Pearson’s χ^2^ test. Multivariable logistic regression models were used to assess the association of each of the lipid metrics expressed as continuous variables with self-reported CVD. Variables that were significantly different between individuals with and without self-reported CVD were considered confounders and were adjusted for in each of the multivariable logistic regression models. Variables were considered collinear if they had variance inflation factor (VIF)>5.0, but no such events were observed. All tests were considered statistically significant at P<0.05.

## Results

### Characteristics of the study participants

The study participants were categorized under those with and those without self-reported CVD as illustrated in Table 1. The study population was made up of 46% men and 54% women. The proportion of current smokers in the population was 10.82% while that of physically active and obese individuals was 85.75% and 2.66% respectively. The burden of hypertension was 21.70%. The proportion of high SES individuals was greater among those with self-reported CVD than those without self-reported CVD (P = 0.013). Similarly, the average number of meal servings from vendors per month was higher among those with self-reported CVD than those without self-reported CVD (P = 0.013).

**Table 1:** Characteristics of the study population.

Variable	Without self-reported CVD	With self-reported CVD	Total	P value*
Participant numbers	1810	29	1839	-
Age in years [median (IQR)]	51 (46–56)	50 (47–55)	51 (46–56)	0.478
Sex [number (%)]				
Men	832 (45.97)	14 (48.28)	846 (46.00)	0.805
Women	978 (54.03)	15 (51.72)	993 (54.00)	
Current smoking [number (%)]				
No	1613 (89.12)	27 (93.10)	1640 (89.18)	0.443
Yes	197 (10.88)	2 (6.90)	199 (10.82)	
Physical activity [number (%)]				
Normal/high	1554 (85.86)	23 (79.31)	1577 (85.75)	0.317
Low	256 (14.14)	6 (20.69)	262 (14.25)	
Hypertension [number (%)]				
No	1418 (78.34)	22 (75.86)	1440 (78.30)	0.748
Yes	392 (21.66)	7 (24.14)	399 (21.70)	
Obesity [number (%)]				
No	1762 (97.35)	28 (96.55)	1790 (97.34)	
Yes	48 (2.65)	1 (3.45)	49 (2.66)	0.792
Household SES [number (%)]				
Low/normal SES	1424 (78.76)	18 (62.07)	1442 (78.41)	0.031
High SES	386 (21.33)	11 (37.93)	397 (21.59)	
Diet [median (IQR)]				
Fruit Intake (servings/week)	7 (0–7)	7 (0–14)	7 (0–7)	0.956
Vegetable Intake (servings/week)	21 (14–28)	21 (14–21)	21 (14–28)	0.144
Vendor meals (servings/month)	0 (0–60)	30 (0–60)	0 (0–60)	0.013
Glucose (mmol/l) [median (IQR)]	4.5 (4.1–4.9)	4.5 (4.1–4.9)	4.5 (4.1–4.9)	0.901
Blood pressure [median (IQR)]				
DBP (mmHg)	76 (68–84)	78 (69–88)	78 (68–85)	0.430
SBP (mmHg)	121 (109–135)	123 (111–139)	121 (109–135)	0.491
BMI (kg/m^2^) [median (IQR)]	21.0 (19.3–23.1)	21.5 (19.6–25.5)	21.0 (19.3–23.2)	0.133
WC (cm) [median (IQR)]	74 (55–176)	77 (58–108)	74 (55–176)	0.361
HC (cm) [median (IQR)]	86 (62–180)	89 (70–109)	86 (62–180)	0.467
Adipose tissue [median (IQR)]				
SAT (cm)	0.8 (0.6–1.2)	0.9 (0.6–1.8)	0.8 (0.6–1.2)	0.453
VAT (cm)	3.6 (3.0–4.5)	3.9 (3.1–4.8)	3.6 (3.0–4.5)	0.548
Statin use [number (%)]				
No	992 (99.90)	845 (99.90)	1837 (99.90)	
Yes	1 (0.10)	1 (0.10)	2 (0.10)	0.931

*P value is for the comparison of individuals without CVD vs. individuals with CVD; CVD: cardiovascular disease; SES: socioeconomic status; DBP: diastolic blood pressure; SBP: systolic blood pressure; BMI: body mass index; IQR: interquartile range; WC: waist circumference; HC: hip circumference; SAT: subcutaneous adipose tissue; VAT: visceral adipose tissue. Self-reported CVD is self-reported presence of heart failure, stroke or myocardial infarction

The prevalence of self-reported CVD in the total population was only 1.58% whilst that for each of self-reported stroke, heart disease or myocardial infarction was less than 1.0% (Table 2). In participants with self-reported CVD, the most common self-reported CVD endpoint was myocardial infarction (51.7%) followed by stroke (48.3%) and congestive heart failure (3.45%). Only two participants self-reported receiving anti-lipid therapy (Table 1). After running the regression analysis with and without these participants the results remained unchanged. Therefore, these participants were not excluded from the analysis. Median non-HDL-C (P = 0.009), TC/HDL-C (P = 0.003) and LDL-C/HDL-C levels (P = 0.019) of participants with self-reported CVD were significantly higher than the respective levels of those without self-reported CVD (Table 2).

**Table 2:** Self-reported CVD endpoints and median levels of lipid metrics according to self-reported CVD status.

Variable	Without self-reported CVD	With self-reported CVD*	Total	P value**
Participant numbers	1810	29	1839	-
CVD endpoints [number (%)]				
MI	0 (0.00)	15 (51.7)	15 (0.82)	-
Stroke	0 (0.00)	14 (48.3)	14 (0.76)	-
Congestive heart failure	0 (0.00)	1 (3.45)	1 (0.05)	-
Presence of self-reported CVD [number (%)]	0 (0.00)	29 (100)	29 (1.58)	-
Lipid metrics [median (IQR)]				
HDL-C (mmol/l)	1.1 (0.9–1.4)	1.0 (0.9–1.4)	1.1 (0.9–1.4)	0.080
LDL-C (mmol/l)	1.6 (1.2–2.1)	1.8 (1.4–2.2)	1.6 (1.2–2.1)	0.092
TG (mmol/l)	0.6 (0.4–0.7)	0.6 (0.5–0.7)	0.6 (0.4–0.7)	0.305
TC (mmol/l)	3.2 (2.6–3.8)	3.5 (2.9–4.0)	3.2 (2.6–3.8)	0.069
Non-HDL-C (mmol/l)	2.0 (1.6–2.5)	2.4 (1.9–3.0)	2.0 (1.6–2.6)	0.009
TG/HDL-C	0.5 (0.4–0.7)	0.6 (0.5–0.8)	0.5 (0.4–0.7)	0.066
TC/HDL-C	2.9 (2.4–3.5)	3.3 (2.9–3.9)	2.9 (2.4–3.5)	0.003
LDL-C/HDL-C	1.5 (1.1–2.6)	1.8 (1.4–2.4)	1.5 (1.1–2.0)	0.019
Remnant chol (mmol/l)	0.4 (0.1–0.8)	0.7 (0.1–1.0)	0.4 (0.1–0.8)	0.127

Self-reported CVD was defined as the presence of self-reported MI, stroke or congestive heart failure

**P value is for the comparison of individuals without CVD vs. individuals with CVD; CVD: cardiovascular disease; MI: myocardial infarction; HDL-C: high density lipoprotein cholesterol; LDL-C: low density lipoprotein cholesterol; TG: triglyceride: TC: total cholesterol; chol: cholesterol.

**Fig 1:**
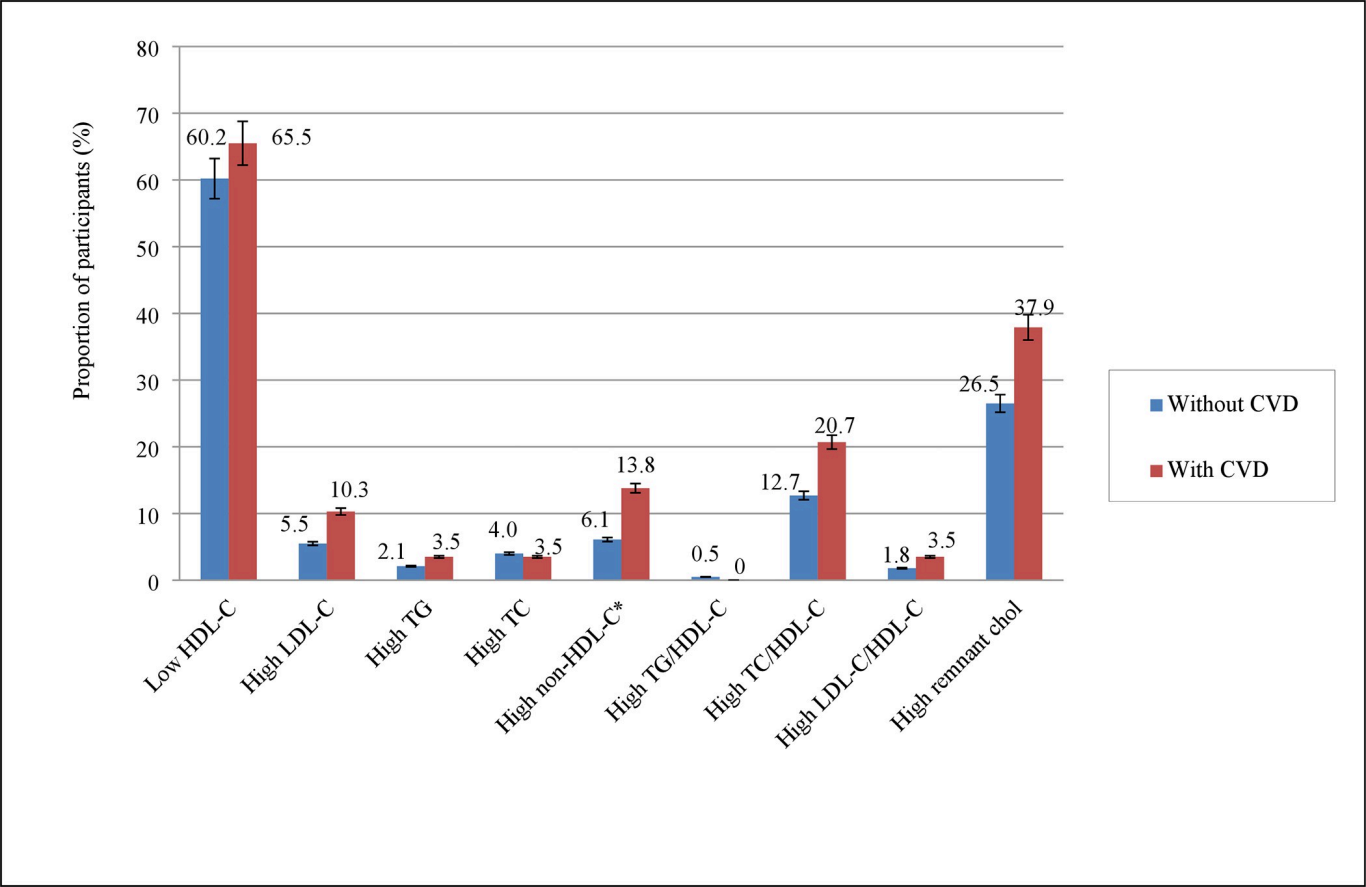
Prevalence of high levels of each of the lipid metrics, and low levels of HDL-C categorized by the presence or absence of reported self-reported CVD in the study participants. *P<0.05; data expressed as % with 95% CIs; CVD: cardiovascular disease; HDL-C: high density lipoprotein cholesterol; LDL-C: low density lipoprotein cholesterol; TG: triglyceride; TC: total cholesterol; chol: cholesterol; CIs: confidence intervals.

Similarly, dyslipidaemia in individuals with and without self-reported CVD were compared. The prevalence of high non-HDL-C was significantly (13.8%) higher among individuals with self-reported CVD than individuals without self-reported CVD (6.1%) (P<0.050) (Fig 1).

### Lipid metrics associated with self-reported CVD in adjusted analyses

Table 3 shows unadjusted and adjusted odds ratios (with 95% CIs) for self-reported CVD for each of the nine lipid metrics in nine separate regression models that were unadjusted or adjusted for SES and meals purchased from vendors. Adjustments were made for these variables because they were the only variables that were found to be significantly different between subjects with and without CVD (see Table 1). In the unadjusted models only non-HDL-C (Table 3, model 5) and LDL-C/HDL-C (Table 3, model 7) were significantly (P = 0.01 and P = 0.02, respectively) associated with CVD. After adjusting for household SES and consumption of vendor meals participants with high non-HDL-C were 58% more likely to develop CVD [(1.58 (1.05, 2.39), P = 0.029] and those with high LDL-C/HDL-C levels were 26% more likely to develop CVD [(1.26 (1.00, 1.59), P = 0.045] compared to subjects with normal levels of these lipid metrics. Consumption of vendor meals was significantly associated with self-reported CVD in all nine regression models whilst SES tended toward a significant association in some of the models with P values ranging from 0.051 to 0.155 (see Table 3).

**Table 3:** Logistic regression analyses of lipid metrics associated with self-reported CVD before and after covariate adjustment.

Model number^a^	Unadjusted models		Adjusted models	
	Independent variable	OR (95% CIs); P value	Independent variables	OR (95% CIs); P value
1	HDL-C	0.49 (0.17, 1.42); 0.191	HDL-C	0.47 (0.16, 1.40); 0.176
			SES	1.07 (1.00, 1.14); 0.051
			Vendor meals	1.22 (1.08, 1.37); 0.001
2	LDL-C	1.42 (0.96, 2.09); 0.078	LDL-C	1.25 (0.84, 1.85); 0.276
			SES	1.06 (0.99, 1.13); 0.094
			Vendor meals	1.21 (1.08, 1.37); 0.002
3	TG	1.12 (0.70, 1.94); 0.561	TG	1.08 (0.54, 2.20); 0.825
			SES	1.06 (1.00, 1.14); 0.060
			Vendor meals	1.22 (1.09, 1.38); 0.001
4	TC	1.40 (0.96, 2.04); 0.081	TC	1.32 (0.91, 1.92); 0.144
			SES	1.06 (0.99, 1.13); 0.101
			Vendor meals	1.23 (1.09, 1.38); 0.001
5	Non-HDL-C	1.72 (1.14, 2.60); 0.010	Non-HDL-C	1.58 (1.05, 2.39); 0.029
			SES	1.05 (0.98, 1.12); 0.155
			Vendor meals	1.22 (1.08, 1.38); 0.001
6	TC/HDL-C	1.14 (0.99, 1.32); 0.059	TC/HDL-C	1.13 (0.98, 1.31); 0.092
			SES	1.06 (0.99, 1.13); 0.074
			Vendor meals	1.22 (1.09, 1.38); 0.001
7	LDL-C /HDL-C	1.30 (1.04, 1.62); 0.020	LDL-C/HDL-C	1.26 (1.00, 1.59); 0.045
			SES	1.06 (0.99, 1.13); 0.089
			Vendor meals	1.22 (1.08, 1.37); 0.001
8	TG/HDL-C	1.09 (0.75, 1.58); 0.640	TG/HDL-C	1.05 (0.69, 1.61); 0.811
			SES	1.07 (1.00, 1.14); 0.058
			Vendor meals	1.22 (1.09, 1.34); 0.001
9	Remnant-C	1.28 (0.76, 2.16); 0.347	Remnant-C	1.35 (0.82, 2.21); 0.238
			SES	1.07 (1.00, 1.14); 0.055
			Vendor meals	1.24 (1.10, 1.39); 0.001

^a^In all models the dependent variable is presence of CVD; HDL-C: high density lipoprotein cholesterol; LDL-C: low density lipoprotein; TG: triglyceride; TC: total cholesterol; CI: confidence interval; SES: socioeconomic status; BMI: body mass index; WC: waist circumference; HC: hip circumference

## Discussion

This study of rural Ghanaian adult men and women investigated the association of a wide array of lipid metrics with self-reported CVD. The results showed that only 1.58% of the population reported a cardiovascular event. The lipid metrics associated with self-reported CVD were non-HDL-C and LDL-C/HDL-C.

The prevalence (1.58%) of self-reported CVD in this rural population was lower than that reported (8.2%) earlier in hospital admissions in urban Ghana [42] and in other studies in SSA [23]. The prevalence of self-reported CVD in this study may be under reported and further studies involving confirmed clinical cases are required to support these findings. However, the low prevalence of both obesity and current smoking and the high level of physical activity reported in this population may partly explain the low level of self-reported CVD events, despite the high prevalence of hypertension. High physical activity, non-smoking and lack of obesity are reported to be associated with healthy cardiovascular outcomes [43].

The strong association of non-HDL-C with self-reported CVD reinforces the recommendation in the latest guidelines for both European [44] and American Cardiology Societies [45] for inclusion of non-HDL-C in the assessment of CVD risk. The observed stronger link of non-HDL-C than several lipid metrics in this study with CVD risk could be due to the representation of all atherogenic apolipoprotein B (ApoB) containing lipoproteins in the non-HDL-C level [10]. Previous findings by Liu et al reported that increased levels of non-HDL-C increased the risk of death among diabetics with acute coronary heart disease and myocardial infarction to a greater extent than did LDL-C, which is considered a primary CVD marker [20]. Other studies in Africans [46] and non-Africans [47, 48] have reported the usefulness of non-HDL-C in CVD risk assessment and as a prognostic marker in CVD treatment. In spite of the low levels of HDL-C reported earlier in this population [26], HDL-C was not associated with self-reported CVD in this study. This is consistent with earlier reports which showed that isolated low HDL-C levels may not necessarily reflect CVD risk [49]. Rather, HDL-C sub-fractions have been reported to improve CVD prediction [50, 51] Future studies are therefore recommended to investigate the relationship of HDL-C sub-fractions with CVD risk in this population.

The observed association of LDL-C/HDL-C ratio with self-reported CVD in all the models is supported by earlier reports of a relationship of this lipid ratio with sudden cardiac death [52] and CVD risk in other populations [53]. It is interesting to note that although high prevalence of low HDL-C was reported in this population [26] HDL-C was not associated with self-reported CVD neither was LDL-C. These data suggest that these individual lipids are not strong markers of CVD but when used in combination become more strongly associated with self-reported CVD. The association of LDL-C/HDL-C with CVD could be attributed to the atherogenic potential of the numerator and the anti-atherosclerotic potential of the denominator acting in combination to provide a stronger association with CVD than that of each lipid species alone [54, 55].

The novelty of this study is that it is the only analysis conducted in SSA of the association of commonly-used lipid metrics with CVD endpoints. This is important because it is known that CVD prevalence is increasing in this region [56, 57] and globally, 80% of deaths related to CVD occur in low- and middle-income countries [2]. In addition, the INTERHEART study demonstrated that premature acute myocardial infarction occurred more often in countries within SSA than in any of the other 52 countries included in that study [58]. It is therefore important to understand the risk factors associated with CVD in SSA and to develop cheap and effective methods for screening for subjects at high risk for these diseases. Dyslipidaemia is prevalent in SSA [28] and is a well-recognized risk factor for CVD and therefore was the focus of this study.

The strength of this study is that it is the first to evaluate and compare the association of a wide array of lipid metrics with self-reported CVD endpoints in a large adult SSA population. Unlike several studies that derived LDL-C from the Friedewald equation, which has several limitations [59], a direct measurement method was used and stringent quality control performed in this study. However, the study was not without limitations. First, it was a cross-sectional analysis of self-reported CVD and clinical data was not used to capture the occurrence of CVD events. Therefore, causal relationships could not be proven. Furthermore, only a small number of participants with self-reported CVD were identified in the study and this reduces the power of the analysis. There is also a shortcoming in the lack of data on apolipoproteins [60] and HDL-C subfractions [50, 51] which are known to be more reliable indicators of CVD risk. Current smoking, physical activity, SES, CVD and diet variables were self-reported and could be prone to bias.

## Conclusion

This is the first study in SSA to assess the association of a wide array of lipid metrics with self-reported CVD. The results suggest a low CVD burden and that non-HDL-C and LDL/HDL-C were associated with CVD and could be used as indicators of CVD risk and prognostic markers of therapy in this study population. Future studies involving longitudinal cohorts and incident cardiovascular cases are recommended to confirm the association of these lipid metrics with CVD.

## Supporting information

S1 Data (XLSX)
